# Emergence of *Cryptosporidium hominis* Monkey Genotype II and Novel Subtype Family Ik in the Squirrel Monkey (*Saimiri sciureus*) in China

**DOI:** 10.1371/journal.pone.0141450

**Published:** 2015-10-28

**Authors:** Xuehan Liu, Na Xie, Wei Li, Ziyao Zhou, Zhijun Zhong, Liuhong Shen, Suizhong Cao, Xingming Yu, Yanchuan Hu, Weigang Chen, Gangneng Peng

**Affiliations:** 1 The Key Laboratory of Animal Disease and Human Health of Sichuan Province, College of Veterinary Medicine, Sichuan Agricultural University, Sichuan Province, 625014, China; 2 The Chengdu Zoo, Institute of Wild Animals, Chengdu, Sichuan Province, 625001, China; Sichuan University, CHINA

## Abstract

A single *Cryptosporidium* isolate from a squirrel monkey with no clinical symptoms was obtained from a zoo in Ya’an city, China, and was genotyped by PCR amplification and DNA sequencing of the small-subunit ribosomal RNA (SSU rRNA), 70-kDa heat shock protein (HSP70), *Cryptosporidium* oocyst wall protein, and actin genes. This multilocus genetic characterization determined that the isolate was *Cryptosporidium hominis*, but carried 2, 10, and 6 nucleotide differences in the SSU rRNA, HSP70, and actin loci, respectively, which is comparable to the variations at these loci between *C*. *hominis* and the previously reported monkey genotype (2, 3, and 3 nucleotide differences). Phylogenetic studies, based on neighbor-joining and maximum likelihood methods, showed that the isolate identified in the current study had a distinctly discordant taxonomic status, distinct from known *C*. *hominis* and also from the monkey genotype, with respect to the three loci. Restriction fragment length polymorphisms of the SSU rRNA gene obtained from this study were similar to those of known *C*. *hominis* but clearly differentiated from the monkey genotype. Further subtyping was performed by sequence analysis of the gene encoding the 60-kDa glycoprotein (gp60). Maximum homology of only 88.3% to *C*. *hominis* subtype IdA10G4 was observed for the current isolate, and phylogenetic analysis demonstrated that this particular isolate belonged to a novel *C*. *hominis* subtype family, IkA7G4. This study is the first to report *C*. *hominis* infection in the squirrel monkey and, based on the observed genetic characteristics, confirms a new *C*. *hominis* genotype, monkey genotype II. Thus, these results provide novel insights into genotypic variation in *C*. *hominis*.

## Introduction

As clinically significant parasites, protozoa of the genus *Cryptosporidium* infect epithelial cells in the microvillus border of the gastrointestinal tract in a broad range of vertebrates and have a worldwide distribution. *Cryptosporidium* causes self-limiting diarrhea in immunocompetent individuals, whereas the infection is usually chronic and life-threatening in immunocompromised hosts, in which effective treatments are currently unavailable [1, 2]. *Cryptosporidium* oocysts are ubiquitous in the environment and commonly employ a variety of transmission routes, including direct contact with infected patients or animals and intake of contaminated food or water, to cause cryptosporidiosis of different hosts, with an incubation period of approximately 7 days [3].

Currently, molecular biological techniques are generally used for detecting and differentiating *Cryptosporidium* parasites at the species/genotype and subtype levels. To date, around 28 known species and more than 70 genotypes have been confirmed based on genotyping data. Molecular epidemiological studies on human cryptosporidiosis indicate that 15 *Cryptosporidium* species (*Cryptosporidium parvum*, *C*. *hominis*, *C*. *meleagridis*, *C*. *felis*, *C*. *canis*, *C*. *muris*, *C*. *suis*, *C*. *andersoni*, *C*. *cuniculus*, *C*. *ubiquitum*, *C*. *fayeri*, *C*. *scrofarum*, *C*. *viatorum*, *C*. *bovis*, and *C*. *erinacei*) and five genotypes (horse genotype, skunk genotype, monkey genotype, chipmunk genotype I, and mink genotype) are infectious to humans [4–10]. Of these, *C*. *hominis* and *C*. *parvum* are responsible for over 90% of clinical cases. Although *C*. *hominis* primarily infects humans, natural infection by the parasite rarely occurs in dugong, cattle, goats, rhesus monkeys, and pigeons. In addition, transmission to a few other mammals can occur [11].

The 60-kDa glycoprotein (gp60) gene, a popular subtyping marker, has been used extensively in studies of *C*. *hominis* and *C*. *parvum* infections in humans and other mammals, because of its sequence heterogeneity and polymorphism. By employing this highly discriminatory marker, subtype identification, tracing of the infection source, and assessment of the transmission dynamics of *C*. *hominis* and *C*. *parvum* can be performed [12]. So far, at least 25 major subtype families, 16 for *C*. *parvum* (IIa to IIp) and nine for *C*. *hominis* (Ia, Ib, and Id to Ij), have been confirmed in various hosts worldwide [9, 13]. These include more than 80 *C*. *parvum* and nearly 80 *C*. *hominis* subtypes, further classified according to the number and type of serine-coding trinucleotide tandem repeats at the 5′ end of the gp60 gene [12, 13].

However, to the best of our knowledge, there is little literature on *Cryptosporidium* infection in monkeys and no reports of transmission to the squirrel monkey in China. In the present study, to further explore the genetic characteristics of *C*. *hominis*, a single isolate from the squirrel monkey was characterized at the molecular level by conducting genotyping and subtyping based on multilocus sequence analysis. Our molecular data indicated that the current isolate was a new variant of *C*. *hominis*, monkey genotype II, and also identified a novel subtype family, IkA7G4.

## Materials and Methods

### Ethical statement

This study was performed according to the Law of the People’s Republic of China on the Protection of Wildlife (modified by Sichuan province, no SC0707304), and was approved by the Giant Panda Breeding Research Foundation (http://www.pandahome.org/en/). No specific permits were required for the described field studies. All feeders at the zoo were required to collect the fresh stool samples of wildlife using disposable polyethylene gloves, in accordance with the Animal Ethics Procedures and Guidelines of the People’s Republic of China; no physical interaction occurred between the feeders and any of the animals. Wildlife were not harmed during the collection process. None of the field studies involved endangered or protected species.

### Sample collection and DNA extraction

A total of 26 fresh fecal samples from various animal species were collected in April 2014 at the zoo in Ya’an city, China. They were screened for *Cryptosporidium* oocysts by bright-field microscopy at 400× magnification and species-specific nested PCR for amplification of a small subunit ribosomal RNA (SSU rRNA) gene fragment of approximately 830 bp. The oocysts were concentrated by Sheather’s sugar flotation technique and stored in 2.5% potassium dichromate solution at 4°C prior to genomic DNA extraction.

Genomic DNA was isolated from each fecal sample using the E.Z.N.A.^®^ Stool DNA Kit (D4015–02; OMEGA Biotek Inc.; USA) according to the manufacturer’s instructions. DNA was eluted in 200μL of the kit Solution Buffer and stored at −20°C prior to use in PCR analysis.

### *Cryptosporidium* genotyping

The species/genotype was established for a *Cryptosporidium*-positive isolate by DNA sequencing of the SSU rRNA locus. To further characterize this isolate, PCR products of approximately 1950, 550, and 1060 bp were amplified for the *Cryptosporidium* oocyst wall protein (COWP), 70-kDa heat shock protein (HSP70), and actin genes respectively. The primers and amplification protocols used for nested PCR of all four loci were as previously described [14–17]. Non-acetylated bovine serum albumin (400 ng/mL; TaKaRa, Dalian, China) was used to neutralize PCR inhibitors in all PCR reactions. All secondary PCR products were examined by 1% agarose gel electrophoresis and visualized after ethidium bromide staining. To produce a specific restriction pattern for *Cryptosporidium* species/genotypes, the PCR products of the SSU rRNA gene were digested with *Ssp*I and *Vsp*I (TaKaRa) for restriction fragment length polymorphism (RFLP) analysis of the products by 2.5% agarose gel electrophoresis. PCR products of the actin gene were cloned into the pMD19-T vector (TaKaRa) according to the manufacturer’s protocol. Recombinant plasmids containing actin gene products were sequenced by BJI Technology Co. (Beijing, China) using vector-specific primers. At least two secondary PCR products for each of the SSU rRNA, HSP70, and COWP genes were directly sequenced without cloning, in both directions, using the secondary PCR primers.

### *Cryptosporidium* subtyping

Subtype identification of the positive isolate was carried out by nested PCR amplification and DNA sequencing analyses of an approximately 850-bp fragment of the gp60 gene. Primers and amplification conditions were adopted from a previous study [18]. The gp60 PCR products obtained were directly sequenced without cloning, in both directions. Sequence accuracy was verified by sequencing two PCR products. The established subtype nomenclature was used to classify gp60 subtypes [12].

### Nucleotide sequence analysis

The nucleotide sequences obtained in this study for each locus were aligned with the corresponding *Cryptosporidium* reference sequences downloaded from the GenBank database, using BLAST (http://blast.ncbi.nlm.nih.gov) and ClustalX software (version 1.83; ftp://ftp-igbmc.u-strasbg.fr/pub/ClustalX/). To support the genotype and subtype classification, phylogenetic analysis at each of the abovementioned loci was performed by use of the PHYLIP software, version 3.64 [19]. Neighbor-joining trees between different sequences were constructed to identify *Cryptosporidium* species and subtypes on the basis of evolutionary distances calculated using the Kimura two-parameter model. The reliability of all topology trees was evaluated by the bootstrap method with 1,000 pseudo-replicates and only values above 50% were reported. Phylograms were drawn in the MEGA 4.0 program, and sequence identity analysis was carried out using the MegAlign program in the DNAstar 6.0 software package. Moreover, to more precisely illuminate the evolutionary status of the current isolate, maximum likelihood analysis was preformed in those loci. Likewise, the bootstrap method with 1,000 pseudo-replicates was employed to assess reliability of all topology trees, and the MEGA 4.0 software was used to draw phylograms.

Nucleotide sequences of the SSU rRNA, HSP70, COWP, actin, and gp60 gene fragments obtained in the present study were deposited in the GenBank database under the accession numbers KP314259–KP314263.

## Results

On performing microscopy analysis and SSU rRNA PCR amplification of all stool samples, only one *Cryptosporidium*-positive isolate was detected, and this was from a squirrel monkey. DNA fragments of 838, 1889, 534, and 1103 bp were successfully amplified from the squirrel monkey isolate (SCSM01) by nested PCR for the SSU rRNA, HSP70, COWP, and actin genes, respectively. RFLP analysis of the SSU rRNA secondary PCR product generated visible bands corresponding to 451, 254, and 109 bp with *Ssp*I, and 560 and 104 bp with *Vsp*I; sequencing analysis of the SSU rRNA gene indicated restriction fragment patterns of 451, 254, 109, 13, and 11 bp (*Ssp*I), and 560, 104, 101, and 73 bp (*Vsp*I). These restriction fragment patterns were close to those reported for *C*. *hominis*, but clearly distinct from those of the monkey genotype and other common *Cryptosporidium* species/genotypes [20].

No nucleotide sequence identical to that of the isolate obtained in this study was found for the SSU rRNA, HSP70, actin, or gp60 gene by BLAST analysis of the Genbank database. In the SSU rRNA gene, the sequence of isolate SCSM01 had 2–6 nucleotide differences (an additional C at nucleotide 438) as compared to other *C*. *hominis* isolates, and shared maximum homology of 99.7% with both *C*. *hominis* (AF159110) and the monkey genotype (AF112569). In contrast, for the HSP70 gene, at least 10 nucleotide changes were observed for isolate SCSM01, dispersed over the entire length of the partial HSP70 gene sequence, compared with the sequences previously reported for *C*. *hominis* isolates and with the monkey genotype. These 10 nucleotide changes were notably more than the three between *C*. *hominis* and the monkey genotype. Likewise, the actin gene sequence obtained in this study contained at least 6 nucleotide substitutions compared with previously reported *C*. *hominis* sequences with maximum identity of 99.4%, which was comparable to that between *C*. *hominis* and the monkey genotype (3 nucleotide substitutions). Interestingly, however, the *Cryptosporidium* isolate in the present study yielded a sequence identical to that of *C*. *cuniculus* and known *C*. *hominis*, including the monkey genotype, for the COWP gene. Due to high homologies in those loci, the parasite was likely to belong to *C*. *hominis*.

**Fig 1:**
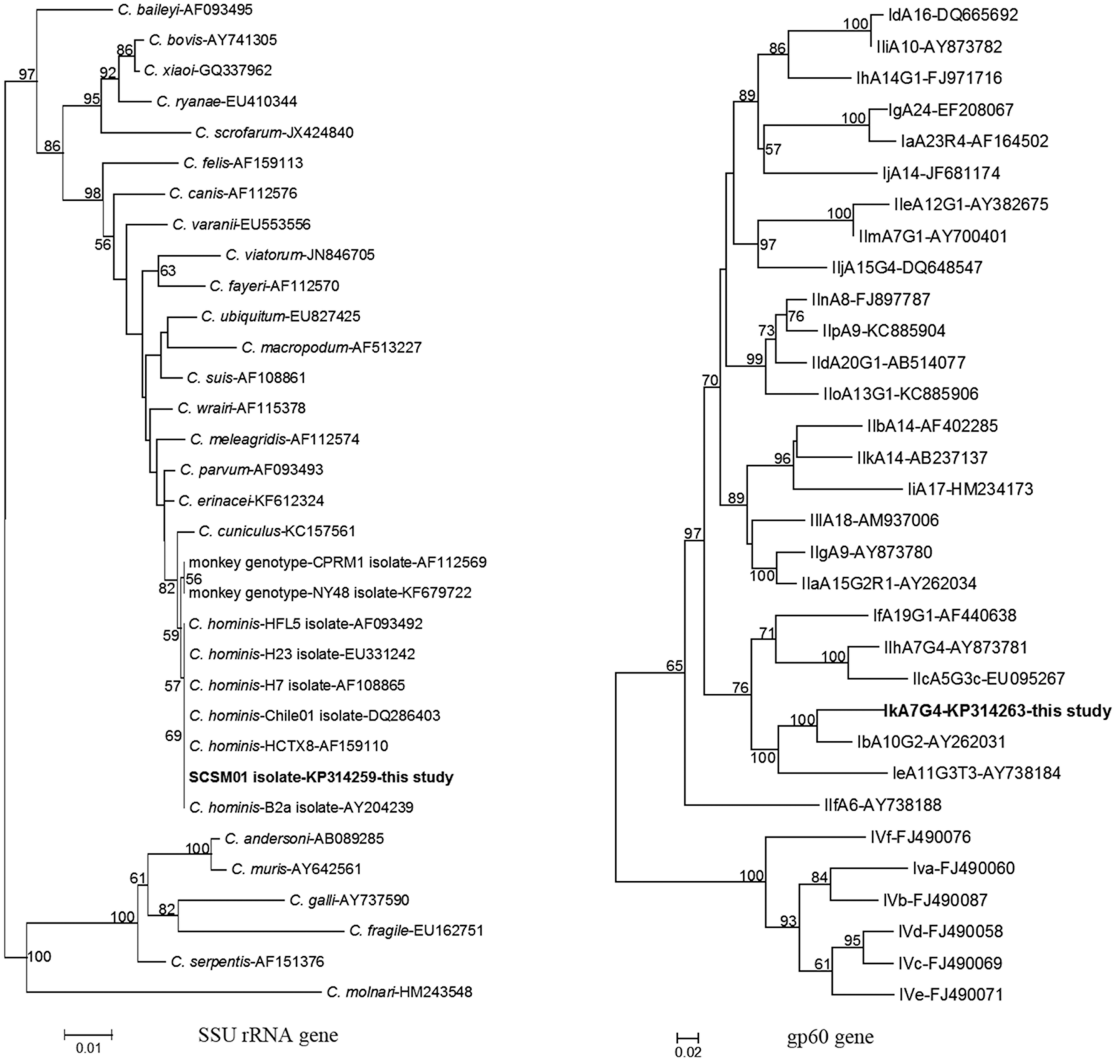
Phylogenetic relationship of the SSU rRNA and gp60 genes sequences of *Cryptosporidium* squirrel monkey isolate in this study to other known *Cryptosporidium* species/genotype and multiple subtype families in *C*. *hominis*, respectively, as inferred by a neighbor-joining analysis based on evolutionary distances calculated using the Kimura two-parameter model. The tree was rooted with partial subtypes of *C*. *fayer* for the gp60 gene. Bootstrap values were obtained using 1,000 pseudo-replicates.

**Fig 2:**
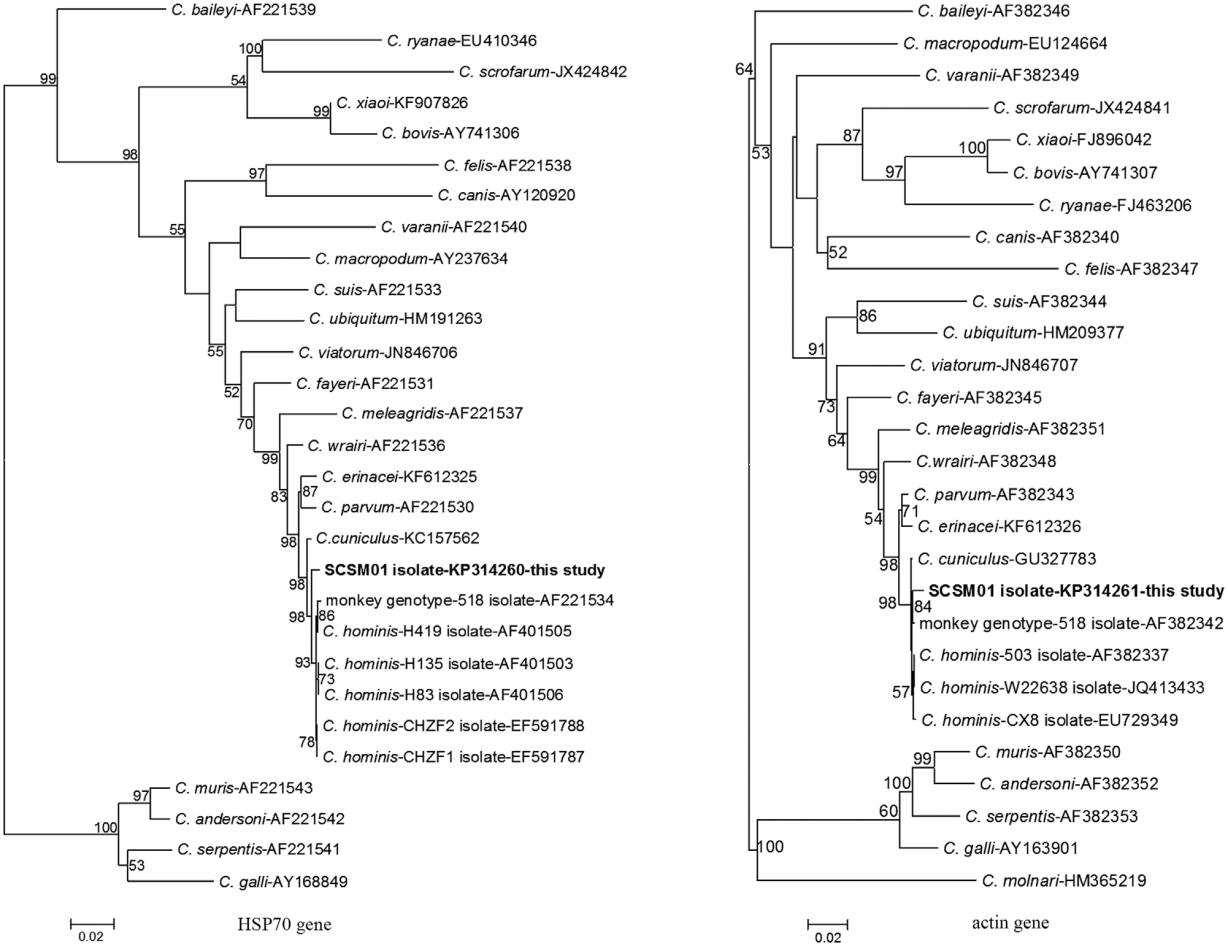
Phylogenetic relationship of *Cryptosporidium* squirrel monkey isolate in this study to other known *Cryptosporidium* species/genotype inferred by neighbor-joining analysis of the HSP70 and actin genes sequences based on evolutionary distances calculated using the Kimura two-parameter model. Bootstrap values were obtained using 1,000 pseudo-replicates.

Phylogenetic trees were constructed for the four genotyped loci of the SCSM01 isolate; inconsistent topologies were produced by the neighbor-joining method. The current isolate and isolates of *C*. *hominis* referenced in the GenBank database, but not the monkey genotype, formed a cluster supported by the bootstrap values in the SSU rRNA and COWP genes, classifying the current isolate as *C*. *hominis* (Fig 1). However, for the HSP70 locus, the isolate from this study formed its own cluster, subsequently grouped together with *C*. *hominis* and the monkey genotype, which was strongly supported by bootstrap analysis (Fig 2). Remarkably, with respect to the actin gene, the squirrel monkey isolate was grouped together with the monkey genotype, which then formed a large cluster with known *C*. *hominis* isolates (Fig 2). In view of the genetic differentiation of this squirrel monkey isolate, whose classification was inconsistent with that of either the reference *C*. *hominis* or the monkey genotype, the existence of a previously unknown *C*. *hominis* genotype, distinct from the monkey genotype, was indicated.

**Fig 3:**
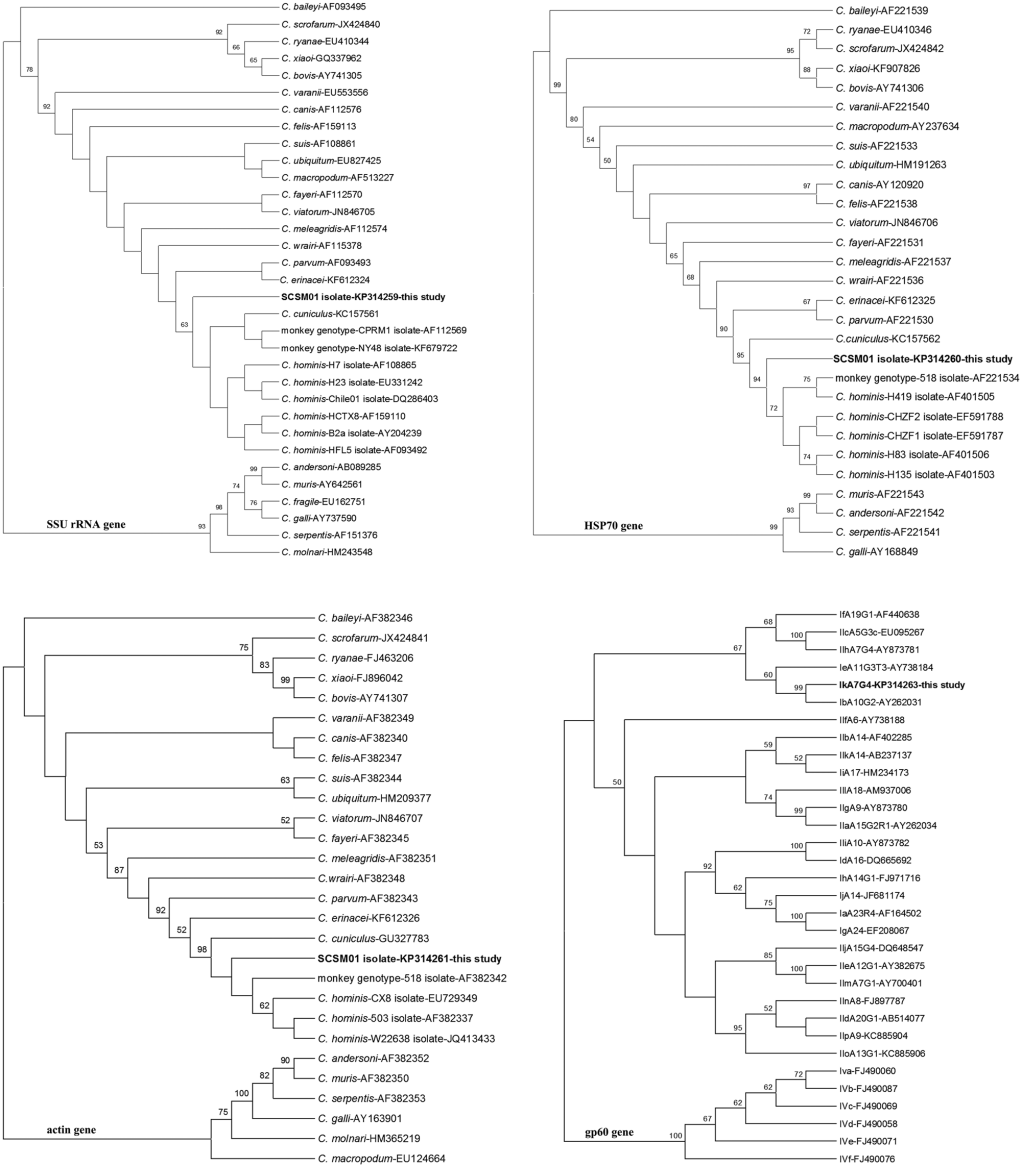
Phylogenetic relationship of the SSU rRNA, HSP70, actin, and gp60 genes sequences of *Cryptosporidium* squirrel monkey isolate detected in this study to other known *Cryptosporidium* species/genotype, respectively, as inferred by a maximum likelihood analysis. Bootstrap values were obtained using 1,000 pseudo-replicates and greater than 50% was shown on nodes.

Additionally, just as expected, similar phylograms occurred by maximum likelihood analysis at each locus compared to the neighbor-joining trees, respectively, and discordant classification was still present among the loci (Fig 3). However, more interestingly, the current isolate SCSM01 evolved alone in advance of monkey genotype and *C*. *hominis* at the phylogenetic level based on maximum likelihood trees in the SSU rRNA and actin genes, differentiating the classifications in neighbor-joining trees. For the SSU rRNA gene, monkey genotype and *C*. *cuniculus* grouped together, and subsequently formed a larger cluster with the known *C*. *hominis*. At the HSP70 locus, the SCSM01 isolate still formed its own cluster in accord with neighbor-joining tree (Fig 3). Therefore, due to the particular phylogenesis, the emergence of a new genotype was demonstrated again.

In a nucleotide BLAST search, an approximately 850-bp fragment of the gp60 gene of isolate SCSM01, obtained in the present study, revealed no known identical sequences. The isolate shared maximum homology of only 88.3% with *C*. *hominis* subtype IdA10G4, which was far lower than that between the known *C*. *hominis* subtypes, suggesting that this particular isolate may belong to a novel subtype family, Ik. Phylogenetic analysis of the gp60 gene sequences showed that the novel subtype family formed a cluster with *C*. *hominis* subtype family Ib and was related to subtype family Ie. Based on currently available molecular data, a novel *C*. *hominis* subtype family, Ik, has been identified and named “IkA7G4” in accordance with current nomenclature conventions [12] (Figs 1 and 3).

## Discussion

*Cryptosporidium hominis* is an important zoonotic agent and has a relatively narrow host spectrum but wide geographical distribution, accounting for approximately 50% of human cryptosporidiosis cases worldwide [12, 21]. *C*. *hominis* infection has been documented in a few species of monkeys, including the rhesus monkey, baboon, slow loris, François’ leaf monkey, and cynomolgus monkey [22–25]. Although 49 squirrel monkeys in China were previously surveyed for *Cryptosporidium* oocysts by genotyping tools, negative results were obtained [22]. However, the present study revealed that *Cryptosporidium* is infective in the squirrel monkey, and *C*. *hominis* maybe harbor a bigger host range.

Currently, various molecular tools have contributed to accurately characterize *Cryptosporidium* species and genotypes from a variety of hosts and different environments samples in genetics. The SSU rRNA, HSP70, COWP, and actin genes, as much conserved markers, are common genetic tools, and their use is typically conclusive in differentiating species/genotype and defining their biological characteristics. By evaluating nucleotide sequence variations and phylogenetic relationships, a few distinct genotypes have been determined, such as the bear, giant panda, ferret, and mink genotypes [7, 26–28]. In addition to having an identical sequence to *C*. *hominis* for the COWP marker gene, the monkey genotype was previously recognized in the rhesus monkey with minor nucleotide substitutions compared to *C*. *hominis* in the SSU rRNA, HSP70, and actin genes (2, 3, and 3 nucleotides, respectively). In the present study, all four of the abovementioned genes were successfully amplified from the SCSM01 isolate. Similar to the findings for the monkey genotype, sequence diversity was observed in the SSU rRNA, HSP70, and actin genes (2, 10, and 6 nucleotide substitutions) when compared to *C*. *hominis* isolates. However, the squirrel monkey isolate was found to contain more single nucleotide polymorphisms in these genes than the monkey genotype (2, 3, and 3 nucleotide substitutions) when both were compared with *C*. *hominis*. These results suggest that the *Cryptosporidium* isolate obtained in this study was likely to represent a new genotype, and are highly consistent with the results of phylogenetic analyses of the SSU rRNA, HSP70, and actin genes on the two methods (neighbor-joining and maximum likelihood). In addition, unique RFLP patterns products produced in the present study showed the emergence of a novel genotype. From the genotypic data, high similarity of the isolate RFLP results to *C*. *hominis* suggests that the new genotype is probably derived from *C*. *hominis*. Furthermore, because it was confirmed as *C*. *hominis* on the basis of high homologies to *C*. *hominis* in the SSU rRNA, COWP and actin genes, the isolate from squirrel monkey was considered to be a novel variant of *C*. *hominis* and was named “monkey genotype II.”

There is extensive intraspecific variation in *C*. *hominis* populations. At present, nine major subtype families, Ia, Ib, Id, Ie, If, Ig, Ih, Ii, and Ij, have been identified in humans and animals worldwide through extensive sequence differences in the non-repeat region of the gp60 gene. Among the nine subtype families, six (Ia, Ib, Id, Ie, If, and Ii) were demonstrated to be infective in various monkeys [20, 22–25, 29]. Remarkably, to our knowledge, only two Ii subtypes, IiA17 and IiA14, are infectious to monkeys and, although the monkey genotype can infect humans, it is still not known whether the Ii family is responsible for zoonosis or is a non-human primate subtype, because of the absence of genetic data for both Ii subtypes reported from epidemiological studies in humans [24, 30]. Significantly, in our study, the identification of a novel subtype, IkA7G4, from analysis of isolate SCSM01 augments our knowledge of *C*. *hominis* subtype families, and also suggests that *C*. *hominis* might comprise a broader subtype population structure. However, due to lack of molecular epidemiology studies of clinical *C*. *hominis* isolates in the same geographical area as the present study, the pathogenicity of the novel Ik family remains unclear.

In conclusion, this study is the first to report *Cryptosporidium* infection of a squirrel monkey in China. The molecular characteristics and subtype of the isolate obtained in this study provide further insights into the host range and genetic diversity of *C*. *hominis*. However, the infection sources and transmission dynamics of *C*. *hominis* monkey genotype II remain to be elucidated, and will require further studies in the same geographical area.
